# Immune checkpoint inhibitors in dMMR-MSI-H colorectal cancer: rationale, progress and prospects

**DOI:** 10.3389/fimmu.2026.1828717

**Published:** 2026-07-01

**Authors:** Guangre Xu, Die Liang, Lei Li, Jian Cheng, Xuejun Wang

**Affiliations:** 1 Department of Gastroenterology and Hepatology, Sichuan Provincial People’s Hospital, School of Medicine, University of Electronic Science and Technology of China, Chengdu, China; 2 Department of Traditional Chinese Medicine, Sichuan Provincial People’s Hospital, School of Medicine, University of Electronic Science and Technology of China, Chengdu, China; 3 Department of anorectal surgery, Hospital of Chengdu University of Traditional Chinese Medicine, Chengdu, China; 4 Department of pediatric surgery, Sichuan Provincial People’s Hospital, University of Electronic Science and Technology of China, Chengdu, China

**Keywords:** colorectal cancer, deficient mismatch repair, high microsatellite instability, immune checkpoint inhibitors, immunotherapy, neoantigens

## Abstract

Colorectal cancer (CRC) with a deficient mismatch repair and high microsatellite instability (dMMR–MSI-H) phenotype represents a biologically distinct subtype characterized by elevated tumor mutational burden and strong immunogenicity. These features contribute to their exceptional responsiveness to immune checkpoint inhibitors (ICIs), especially those targeting the PD-1 and CTLA-4 pathways. Although dMMR–MSI-H tumors constitute only a small fraction of CRC cases, their response to ICIs has redefined therapeutic standards. In this review, we discuss the immunological underpinnings that render these tumors susceptible to immune modulation, summarize key clinical trial findings, and analyze emerging resistance mechanisms. Furthermore, we highlight the evolving landscape of predictive biomarkers and ongoing efforts to increase treatment efficacy through combination strategies and biomarker-driven approaches.

## 1 Introduction

Colorectal cancer (CRC) is a leading cause of cancer-related morbidity and mortality worldwide (1). Despite progress in systemic therapies that have improved outcomes in patients with metastatic CRC (mCRC), patients with microsatellite stable (MSS) tumors generally derive minimal benefit from immunotherapy (2). A notable exception is the subset of tumors with deficient mismatch repair (dMMR) and high microsatellite instability (MSI-H), which represent a biologically distinct group characterized by genomic instability and enhanced immunogenicity (3).

**Figure 1:**
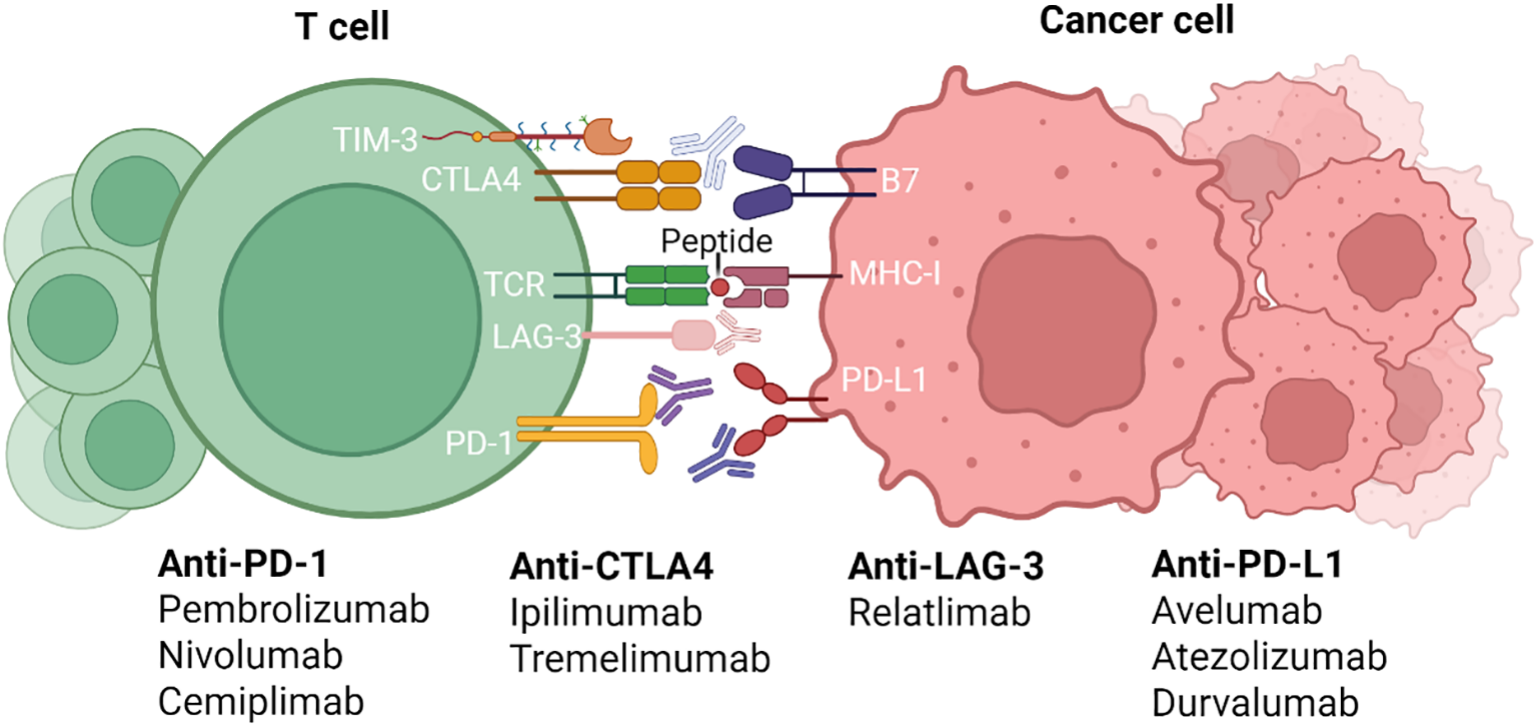
The immune checkpoint pathways between cytotoxic T cells and CRC cells and currently approved ICIs. Inhibitory receptors such as PD-1, CTLA-4, LAG-3, and TIM-3 expressed on activated T cells engage their cognate ligands on tumor or antigen-presenting cells, leading to attenuation of T-cell activation, cytokine production, and cytolytic function. Tumor cells can exploit these immune checkpoints to evade immune surveillance. ICIs disrupt these inhibitory interactions and reinvigorate antitumor immunity. Clinically approved agents include anti–PD-1 antibodies (pembrolizumab, nivolumab, and cemiplimab), anti-PD-L1 antibodies (avelumab, atezolizumab, and durvalumab), anti-CTLA-4 antibodies (ipilimumab and tremelimumab), and anti-LAG-3 antibodies (relatlimab).

Defects in the DNA mismatch repair (MMR) system disrupt the correction of replication errors, leading to an accumulation of insertion–deletion mutations within repetitive microsatellite regions across the genome. This molecular phenotype, known as microsatellite instability, arises from inactivation of key MMR proteins, either through inherited mutations or via epigenetic silencing (4). The resulting hypermutated tumor landscape fosters the generation of abundant tumor-specific neoantigens, which are crucial for effective immune recognition (5). This high neoantigen load correlates with an inflamed tumor microenvironment characterized by dense infiltration of cytotoxic CD8+ T lymphocytes, elevated expression of interferon-stimulated genes, and increased levels of immune checkpoint molecules such as PD-1, PD-L1, and CTLA-4 (6) (**Figure 1**).

In contrast to the “immune-cold” phenotype typically observed in MSS CRCs, dMMR–MSI-H tumors possess a “hot” immune contexture that renders them particularly susceptible to immune checkpoint blockade (7). Clinically, dMMR–MSI-H tumors account for approximately 4–5% of mCRC cases but represent a disproportionately responsive subgroup to immune checkpoint inhibitors (ICIs) (8). The interplay of their molecular instability and immunogenic microenvironment underpins the remarkable success of ICIs in this setting, transforming the therapeutic landscape for these patients (9). However, challenges remain in optimizing treatment strategies, understanding resistance mechanisms, and refining biomarker-driven patient selection.

While prior reviews have largely focused on clinical outcomes or individual biological features of dMMR–MSI-H CRC, this review aims to provide a mechanistically integrated and translationally oriented framework. Specifically, we (i) connect genomic instability, neoantigen landscape, antigen presentation, and interferon signaling into a unified model of ICI responsiveness and resistance; (ii) bridge tumor-intrinsic alterations with tumor microenvironmental and systemic factors, including spatial immune organization, circulating tumor DNA dynamics, and the gut microbiome; and (iii) highlight biomarker-driven and adaptive therapeutic strategies to guide precision immunotherapy. By aligning mechanistic insights with clinical application, this review seeks to advance a more translationally actionable understanding of ICI therapy in dMMR–MSI-H CRC.

## 2 dMMR–MSI-H CRC

**Figure 2:**
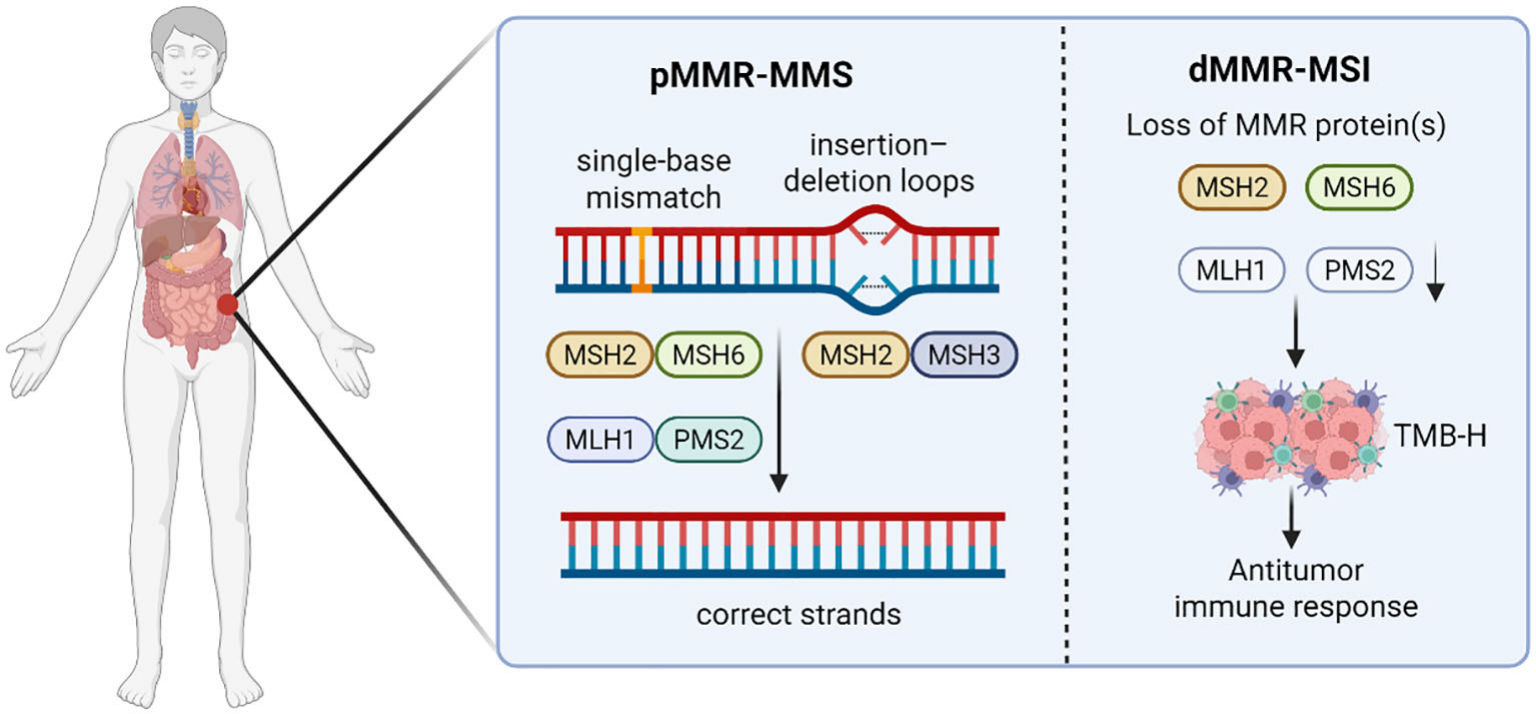
The molecular basis and immunological consequences of MMR proficiency and deficiency in CRC. In proficient MMR (pMMR) tumors, the MMR machinery recognizes and repairs single-base mismatches and insertion–deletion loops during DNA replication, thereby maintaining genomic stability. In contrast, deficient MMR (dMMR) tumors result from the loss or inactivation of one or more MMR proteins, such as MLH1, MSH2, MSH6, and PMS2, leading to microsatellite instability (MSI) and the accumulation of insertion–deletion mutations, particularly in repetitive DNA sequences. The resulting high tumor mutational burden (TMB-H) generates abundant neoantigens that promote immune cell infiltration and potentiate an antitumor immune response, providing a biological rationale for the clinical efficacy of ICIs in dMMR–MSI tumors.

dMMR-MSI-H CRC constitutes a biologically and clinically distinct subset of colorectal malignancies, defined by the inactivation of key MMR genes, most commonly MLH1, MSH2, MSH6, or PMS2 (8). The MMR pathway is essential for preserving genomic integrity by correcting single-base mismatches and small insertion–deletion loops that occur during DNA replication (10). Loss of one or more MMR components, whether through germline mutations, somatic alterations, or epigenetic silencing, such as MLH1 promoter hypermethylation, renders this proofreading system ineffective (11). As a result, short tandem repeats known as microsatellites acquire length variations, giving rise to the molecular phenotype termed MSI (**Figure 2**).

### 2.1 Biology and etiology

MSI can generate a cascade of genomic alterations, particularly when insertions or deletions occur within the coding sequences of key regulatory genes, resulting in frameshift mutations (12). These mutations frequently produce truncated proteins with novel C-terminal peptide sequences not present in normal cells. These altered proteins can serve as tumor-specific neoantigens that are readily recognized by the host immune system (13). This process results in a hypermutated tumor phenotype, often with a tumor mutational burden (TMB) exceeding 20 mutations per megabase (14). The immunological consequence of this genomic instability is profound: MSI-H tumors are typically infiltrated by large numbers of CD8^+^ cytotoxic T lymphocytes and helper T cells, and they often exhibit tertiary lymphoid structures within or adjacent to the tumor mass. The cytokine profiles of these tumors indicate high interferon-γ activity, and immune gene expression signatures reflect an activated or “hot” immune microenvironment (3). Paradoxically, this robust immune activation is frequently counterbalanced by the upregulation of inhibitory immune checkpoint pathways, including PD-1, PD-L1, CTLA-4, LAG-3 and TIM-3 (15). This adaptive resistance state allows tumor cells to evade immune destruction despite their high antigenicity and provides a mechanistic rationale for the observed sensitivity of MSI-H tumors to ICIs, which can restore effective antitumor immune responses by blocking these inhibitory signals (16).

From an etiological perspective, dMMR–MSI-H CRCs can be broadly classified into sporadic and hereditary cases (3). Sporadic MSI-H tumors most often result from MLH1 promoter hypermethylation, leading to transcriptional silencing of the MLH1 gene, and are commonly associated with the BRAF V600E mutation (17). This subtype predominates in older patients, particularly women, and is frequently accompanied by the CpG island methylator phenotype (CIMP-high) (18). In contrast, hereditary cases typically arise from germline pathogenic variants in MMR genes, a condition referred to as Lynch syndrome (19). Lynch-associated MSI-H tumors present at younger ages, affect both sexes equally, and are associated with a markedly elevated lifetime risk of not only CRC but also endometrial, ovarian, gastric, urinary tract, and other malignancies (20). Unlike sporadic MSI-H cancers, Lynch syndrome tumors rarely harbor BRAF V600E mutations or MLH1 promoter methylation (21).

### 2.2 Diagnostic testing

The accurate determination of the MSI-H/dMMR status is critical for patient management, with implications for prognosis, therapeutic selection, and hereditary cancer risk assessment (22). Universal testing of all newly diagnosed CRCs for MSI or MMR status is recommended by major guidelines, including the National Comprehensive Cancer Network (NCCN) and the European Society for Medical Oncology (ESMO) (23). Three main laboratory methods are employed in clinical practice: immunohistochemistry (IHC), PCR-based MSI testing, and next-generation sequencing (NGS) (24). IHC was used to detect the presence or absence of nuclear expression of the four core MMR proteins (MLH1, MSH2, MSH6, and PMS2) in tumor cells, and the surrounding normal tissue was used as an internal control (25). Loss of expression indicates functional deficiency of the corresponding protein, and the specific pattern of protein loss often suggests the gene involved. For example, the concurrent loss of MLH1 and PMS2 usually reflects MLH1 inactivation, whereas the concurrent loss of MSH2 and MSH6 implicates MSH2 (26). IHC is rapid, widely available, and relatively inexpensive, making it a common first-line approach. However, it may miss certain nontruncating mutations that preserve antigenicity and thus yield false-negative results. PCR-based MSI testing evaluates a defined panel of microsatellite markers, typically mononucleotide and dinucleotide repeats, and compares their lengths in tumor DNA with those in matched normal DNA (27). The widely used Bethesda panels include BAT25, BAT26, D2S123, D5S346, and D17S250, whereas the pentaplex mononucleotide panels (BAT25, BAT26, NR21, NR24, MONO27) offer high specificity (28). PCR directly measures the functional consequences of MMR loss but requires adequate tumor cellularity and paired normal tissue for optimal accuracy. NGS-based methods, which are increasingly adopted in clinical oncology, can computationally determine MSI status by analyzing hundreds to thousands of microsatellite loci genome wide without the need for matched normal DNA (29). In addition, NGS allows simultaneous assessment of TMB; actionable driver mutations such as those in KRAS, NRAS, BRAF, and PIK3CA; and other biomarkers relevant to treatment selection (30). While more expensive and resource intensive, NGS is valuable when tissue availability is limited, when the IHC and PCR results are discordant, or when a comprehensive molecular profile is desired.

### 2.3 Epidemiology and clinical implications

Epidemiologically, MSI-H status is found in approximately 12–15% of all newly diagnosed CRC cases, but the prevalence declines sharply in the metastatic setting, where only 4–5% of cases are MSI-H (31). This reduction reflects a biological tendency toward less aggressive dissemination; compared with MSS tumors, MSI-H tumors appear to have a lower propensity for hematogenous metastasis (32). When metastases occur, MSI-H mCRC is less likely to involve the liver and more likely to spread to peritoneal surfaces or distant lymph nodes (33). Clinically, MSI-H tumors tend to arise in the proximal colon, exhibit mucinous or poorly differentiated histology, and present with conspicuous lymphocytic infiltration and Crohn’s-like peritumoral lymphoid aggregates (34). These features, combined with demographic trends, such as the predominance of sporadic MSI-H cases in elderly women, allow for some degree of clinical suspicion, but molecular testing remains essential for definitive classification. The integration of biological understanding, epidemiological context, and robust diagnostic testing has direct clinical implications. In the metastatic setting, the unique immune biology of dMMR–MSI-H tumors makes them highly sensitive to PD-1–based ICIs, which are now preferred first-line therapies in this population on the basis of trials (35, 36). In localized disease, the MSI-H status serves as a prognostic biomarker and informs adjuvant chemotherapy decisions, particularly the lack of benefit from the single agent 5-fluorouracil in stage II MSI-H tumors (37). Moreover, accurate identification of Lynch syndrome enables targeted cancer prevention strategies for patients and at-risk relatives through enhanced surveillance and prophylactic measures (38, 39). In summary, dMMR–MSI-H mCRC represents a rare but clinically crucial molecular subtype of CRC, defined by distinctive genetic alterations, immunogenic tumor biology, and epidemiological patterns. Comprehensive and precise testing is indispensable, ensuring that patients are appropriately selected for immune-based therapies and that hereditary cancer syndromes are recognized in time to alter the disease course for both patients and their families.

## 3 ICIs in dMMR–MSI-H CRC

dMMR–MSI-H CRC is a biologically distinct subgroup that has demonstrated exceptional responsiveness to ICIs. This responsiveness arises from the unique immunobiology of dMMR–MSI-H tumors, which harbor high TMB and generate abundant tumor-specific neoantigens due to the accumulation of insertion–deletion mutations in microsatellite regions (40). These neoantigens are recognized by the host immune system, resulting in a highly inflamed tumor microenvironment characterized by dense infiltration of CD8^+^ cytotoxic T lymphocytes, activated CD4^+^ helper T cells, and antigen-presenting dendritic cells (41). However, these tumors also exhibit adaptive immune resistance, with upregulation of inhibitory immune checkpoints (42, 43). ICIs target these inhibitory pathways, reinvigorate exhausted T cells and restore antitumor immune responses, often leading to durable clinical benefits.

### 3.1 Clinical treatment

The clinical development of ICIs for dMMR–MSI-H CRC has advanced rapidly over the past decade, reshaping treatment standards (**Table 1**). The first clinical signals of ICI activity in this setting emerged from early-phase studies of PD-1 blockade (44, 45). These studies demonstrated a striking contrast in objective response rates (ORRs), along with durable disease control in many responders. These findings led to the hypothesis that the MSI status could serve as a robust predictive biomarker for ICI efficacy. Subsequent larger, multicohort trials confirmed these early observations and established the foundation of the current treatment paradigm (46).

**Table 1:** Landmark clinical trials of ICIs in dMMR–MSI-H CRC.

Treatment setting	Clinical trial	Phase	Intervention	Sample size	ORR	PFS	OS	Key notes	Ref
First-line	NCT02563002	III	Pembrolizumab vs chemo	Untreated dMMR–MSI-H mCRC (n=307)	43.8%	16.5 mo vs 8.2 mo	24-mo OS: 81% vs 65%	Practice-changing, established pembrolizumab as standard first-line therapy	(48, 174, 175)
	NCT04008030	III	Nivolumab ± Ipilimumab vs chemo or nivolumab	Untreated and previously treated dMMR–MSI-H mCRC (n≈830)	Combo superior to nivolumab for PFS	HR ~0.62; higher 12-, 24-, 36-mo PFS rates with combo	Pending mature OS	Randomized data support dual checkpoint across lines	(144)
Refractory	NCT02460198	II	Pembrolizumab	Refractory dMMR–MSI-H mCRC (n≈124 across cohorts)	33–34%	Median PFS ~4 mo, durable disease control in responders	Median OS ~31 mo	Confirmed pembrolizumab activity in heavily pretreated patients	(49, 176)
	NCT02060188	II	Nivolumab ± Ipilimumab	Refractory dMMR–MSI-H mCRC (n≈119 for mono, 45 for combo)	31% (mono), 55% (combo)	12-mo PFS: 71% (combo)	12-mo OS: 83% (combo)	Demonstrated robust activity of dual checkpoint blockade with manageable safety profile	(36, 52, 53)
	NCT03186326	II	Avelumab vs physician’s-choice chemo (2 L)	dMMR–MSI-H mCRC after 1 prior line (n≈122)	~19–30% (avelumab)*	Improved PFS vs chemo	OS maturing	First randomized PD-L1 vs chemo in 2 L; disease control improved with avelumab	(177, 178)
	NCT03150706	II	Avelumab monotherapy	Previously treated dMMR–MSI-H mCRC (n≈33)	~24%	Median PFS ~3–4 mo	NR	Early evidence of PD-L1 activity in MSI-H mCRC	(179)
Tissue-agnostic	NCT01876511	II	Pembrolizumab	dMMR CRC & non-CRC (n≈41, incl. 11 CRC)	40% (CRC)	NR	NR	First proof-of-concept, led to tissue-agnostic FDA approval consideration	(35)
	NCT02715284	I/II	Dostarlimab	dMMR solid tumors incl. mCRC (n≈209 overall, ~69 CRC)	41–44% (overall dMMR)	Median PFS ~6.9 mo	NR	Led to FDA accelerated approval for dMMR tumors; includes mCRC cohort	(180)
	NCT03736889	II	Tislelizumab	MSI-H/dMMR solid tumors incl. mCRC (n≈113 overall, ~35 CRC)	~40–45% (overall dMMR)	Durable disease control	NR	Confirms class effect; CRC subset included although not powered for CRC-specific estimates	(181)

One of the most influential studies is a randomized phase III trial comparing pembrolizumab with standard chemotherapy in previously untreated patients with dMMR–MSI-H CRC (47, 48). Pembrolizumab demonstrated a median progression-free survival (PFS) of 16.5 months versus 8.2 months with chemotherapy, corresponding to a hazard ratio (HR) of 0.60, and achieved an ORR of 43.8% (47). The responses were notably durable, with the median duration of response not reached at the time of analysis. Importantly, pembrolizumab was associated with fewer grade 3–4 treatment-related adverse events (22% vs 66%) and better preservation of health-related quality of life, underscoring its clinical advantage not only in efficacy but also in tolerability. These findings establish pembrolizumab as the preferred first-line therapy for dMMR–MSI-H CRC according to international guidelines. For patients who have received prior therapy, the phase II KEYNOTE-164 trial confirmed the robust activity of pembrolizumab in the refractory setting, reporting durable responses in heavily pretreated dMMR–MSI-H CRC patients (49). Similarly, nivolumab, another PD-1 inhibitor, demonstrated substantial clinical benefit in the single-arm phase II CheckMate 142 study, which enrolled patients with previously treated dMMR–MSI-H mCRC (50, 51). Nivolumab monotherapy achieved an ORR of 31% and a disease control rate of 69% at 12 weeks, with manageable toxicity profiles. Given that CTLA-4 blockade may potentiate PD-1 inhibition by broadening T-cell priming and activation, a combination regimen of nivolumab plus low-dose ipilimumab was also evaluated in CheckMate 142 (52). In previously treated patients, the combination yielded an ORR of 55% and a 12-month PFS rate of 71%, with manageable immune-related adverse events. Importantly, the combination was later tested in the first-line setting within the same study framework, showing an ORR of 69% and 12-month PFS and overall survival (OS) rates of 77% and 83%, respectively, suggesting a potentially higher initial tumor control rate than PD-1 monotherapy (36, 53). While direct head–to-head comparisons are lacking, these data raise important considerations about whether combination therapy may benefit certain patients—such as those with high tumor burden or aggressive disease—by inducing faster and deeper responses. Mechanistically, the sustained benefit of ICIs in patients with dMMR–MSI-H CRC is attributed to multiple immune processes. PD-1 blockade reinvigorates exhausted effector T cells within the tumor microenvironment, restores cytotoxic activity, and supports the proliferation of tumor-specific clones (54). CTLA-4 inhibition acts earlier in the immune activation cascade, promoting T-cell priming and expanding the diversity of the antitumor T-cell repertoire (55). The presence of tertiary lymphoid structures, increased interferon-γ signaling, and high baseline PD-L1 expression further contribute to ICI responsiveness (56). Moreover, the high intratumoral mutational load facilitates the generation of multiple nonoverlapping neoantigens, reducing the likelihood of complete immune escape through antigen loss variants.

### 3.2 ICI resistance

**Figure 3:**
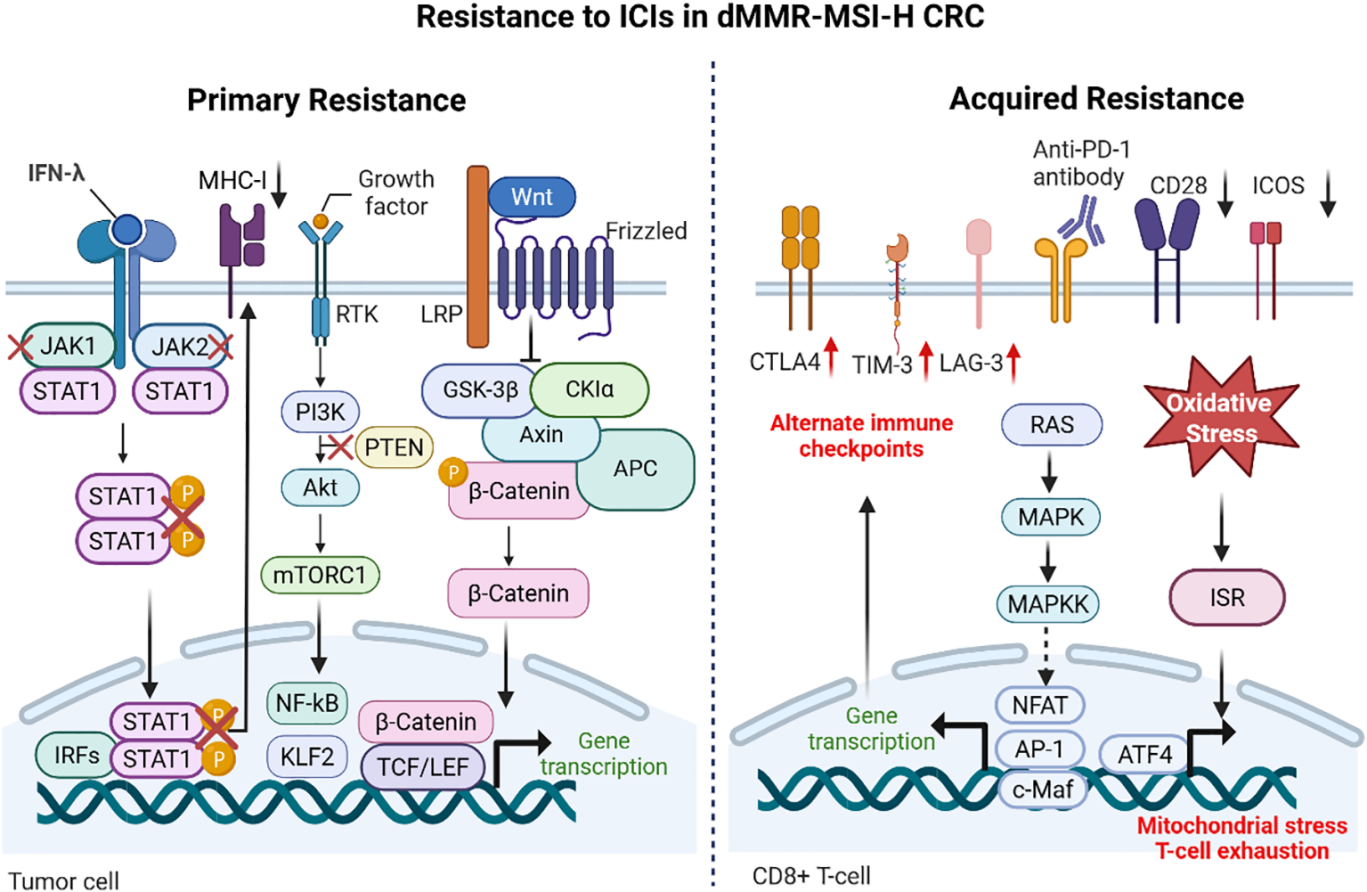
Mechanistic overview of primary and acquired resistance to ICIs in dMMR–MSI-H CRC. Left panel: Primary resistance arises from tumor-intrinsic alterations that impair immune recognition and signaling. Loss-of-function mutations in JAK1/2 abrogate downstream STAT1 activation and disrupt interferon (IFN)-mediated signaling and antigen presentation via MHC-I. Activation of the PI3K–AKT–mTORC1 axis or constitutive Wnt/β-catenin signaling represses T-cell infiltration by modulating the expression of transcriptional regulators such as NF-κB and TCF/LEF. Right panel: Acquired resistance develops during therapy through adaptive immune remodeling. Upregulation of alternative inhibitory receptors, including CTLA-4, TIM-3, and LAG-3, attenuates T-cell activation despite PD-1 blockade. Sustained MAPK signaling, oxidative stress and activation of the integrated stress response (ISR) promote transcriptional reprogramming via NFAT, AP-1, ATF4, and c-Maf, driving CD8^+^ T-cell exhaustion and loss of cytotoxicity.

Despite the highly immunogenic nature of dMMR–MSI-H CRC, a clinically meaningful subset of tumors exhibits primary resistance (**Table 2**), while others develop acquired resistance after an initial response, highlighting that TMB alone is insufficient to guarantee effective immune control (**Figure 3**).

**Table 2:** Immunological subsets associated with response and resistance to ICI in dMMR–MSI-H CRC.

Immune subset	Key functional features	Association with ICI response	Contribution to resistance	Ref
CD8^+^ cytotoxic T cells	Tumor antigen recognition; perforin/granzyme-mediated killing; IFN-γ production	High infiltration and activation strongly correlate with durable responses	Functional exhaustion (PD-1^high^, TIM-3^+, LAG-3^+) limits tumor clearance	(182)
CD4^+^ Th1 cells	Support CD8^+^ T cell priming; IFN-γ secretion; enhancement of antigen presentation	Promote robust anti-tumor immunity and ICI responsiveness	Reduced Th1 polarization weakens effective immune activation	(183)
Tregs	Immunosuppression via IL-10, TGF-β; inhibition of effector T cells	Low Treg infiltration favors response	Enrichment suppresses CD8^+^ T cell activity and promotes immune evasion	(184)
Exhausted T cells	Chronic antigen exposure; high inhibitory receptor expression	Early/progenitor exhausted T cells may be reinvigorated by ICIs	Terminal exhaustion states are refractory to reinvigoration, limiting response	(185)
Dendritic cells (DCs)	Antigen presentation; T cell priming; cross-presentation	Functional DCs enhance neoantigen presentation and T cell activation	Impaired maturation or antigen presentation reduces effective priming	(186)
Tumor-associated macrophages (TAMs)	Plasticity between M1 (pro-inflammatory) and M2 (immunosuppressive) states	M1-like macrophages support anti-tumor immunity	M2 polarization promotes T cell suppression, angiogenesis, and tumor progression	(187)
Myeloid-derived suppressor cells (MDSCs)	Suppress T cell function via arginase, ROS, and nitric oxide	Low levels are associated with better response	Expansion inhibits T cell activation and promotes resistance	(188)
Natural killer (NK) cells	MHC-independent cytotoxicity; cytokine production	Contribute to early tumor control and immune priming	Dysfunction reduces innate immune contribution	(189)
B cells/tertiary lymphoid structures (TLS)	Antibody production; antigen presentation; organization of TLS	Presence of TLS correlates with improved ICI response	Absence or disorganization of TLS is linked to weaker immune activation	(190)
Neutrophils (TANs)	Functional plasticity (anti- vs pro-tumor phenotypes)	Limited direct association with response	Pro-tumor (N2-like) neutrophils promote immunosuppression and metastasis	(191)

Primary resistance to ICIs in in dMMR–MSI-H CRC largely arises from defects in tumor antigen presentation and interferon-γ signaling. Truncating mutations, deletions, or loss of heterozygosity in β2-microglobulin (B2M), as well as loss of HLA class I heavy chains or impaired peptide processing via TAP1/2, ERAP1/2, and NLRC5 downregulation, disrupt MHC-I surface expression and limit CD8^+^ T-cell recognition (57–60). WNT/β-catenin activation creates a dendritic-cell desert and impairs T-cell priming, the TGF-β-rich stroma establishes an exclusionary barrier that keeps lymphocytes at the invasive margin, and oncogenic signaling through PI3K/AKT or MAPK can drive immunosuppressive cytokine programs (61). Beyond these discrete genetic lesions, resistance is shaped by layered qualitative and microenvironmental constraints. Even with high tumor mutational burden, suboptimal neoantigen quality, clonal heterogeneity, and weak MHC binding can reduce immunogenicity (62–64). Oncogenic pathways such as WNT/β-catenin, TGF-β signaling, and PI3K/AKT or MAPK activation further remodel the tumor microenvironment by limiting dendritic cell recruitment and promoting T-cell exclusion or suppression. These effects are amplified in specific anatomical contexts, such as liver metastases, which induce systemic immune tolerance via Kupffer cells and sinusoidal endothelial cells (33).

In parallel, the tumor microenvironment (TME) of some MSI-H lesions skews toward immunosuppression, with abundant regulatory T cells (Tregs), M2-polarized tumor-associated macrophages, and myeloid-derived suppressor cells (MDSCs) producing arginase, nitric oxide, and reactive oxygen species that blunt T-cell function (6, 22). Metabolic constraints such as hypoxia, glucose deprivation, lactate accumulation, IDO1-mediated tryptophan depletion, and CD39/CD73-driven adenosine production contribute to the functional exhaustion of infiltrating lymphocytes (65, 66). Additional extrinsic influences arise from the gut microbiome, where antibiotic exposure, reduced microbial diversity, or loss of immunostimulatory taxa such as *Akkermansia* and *Bifidobacterium* have been linked to diminished ICI activity, likely through impaired antigen cross-presentation and altered mucosal immunity (67).

Importantly, the mechanistic insights described above have been partially interrogated using a range of preclinical systems, including mismatch repair-deficient syngeneic mouse models, GEMMs, patient-derived xenografts (PDXs) and organoid-based co-culture platforms, as well as ex vivo tumor–immune slice cultures (68). These models collectively enable functional dissection of immune editing, antigen presentation loss, and interferon-γ pathway disruption, while also allowing controlled evaluation of checkpoint blockade resistance under immune-competent or humanized conditions. However, each system captures only selected components of the human dMMR–MSI-H tumor ecosystem, murine models provide intact immunity but differ in neoantigen repertoire and HLA biology, whereas PDX and organoid systems preserve tumor genomics but lack fully competent immune microenvironments (69). As a result, no single model fully recapitulates the spatial and temporal immune evolution observed in patients. This limitation is mirrored in the clinical setting, where biomarkers such as MSI status, tumor mutational burden, PD-L1 expression, B2M/JAK alterations, and interferon-γ signatures are largely static and correlative, whereas resistance is a dynamic process driven by continuous tumor–immune interaction (70, 71). Consequently, preclinical models provide mechanistic validation rather than direct predictive equivalence to clinical biomarkers, underscoring the need for integrated longitudinal sampling and multi-platform modeling approaches to bridge this translational gap.

In contrast to primary resistance, acquired resistance arises from dynamic tumor evolution under the selective pressure of immune checkpoint blockade. Initial tumor regression is frequently followed by the outgrowth of resistant subclones through immunoediting, in which highly immunogenic, neoantigen-expressing populations are eliminated, leading to the enrichment of less immunogenic or antigen-loss variants. This process typically occurs after an initial period of disease control and is driven by ongoing immune selection within the tumor microenvironment. Over time, tumor clones harboring loss of highly immunogenic frameshift neoantigens or exhibiting HLA class I loss of heterozygosity become preferentially selected, ultimately restoring immune escape despite prior clinical response (72). Upon PD-1 blockade, new truncating mutations in B2M or JAK1/2, which are absent at baseline, have been reported, recreating the defects in antigen presentation and interferon-γ responsiveness observed in primary resistance (73). Tumors may also adapt by increasing the expression of alternative inhibitory checkpoints, such as TIM-3, LAG-3, TIGIT, VISTA, or BTLA, or by amplifying PD-L1, thereby restoring suppressive signaling despite PD-1 inhibition (74). Epigenetic reprogramming also plays an important role. Increased H3K27me3 and DNA methylation can silence genes involved in antigen processing or endogenous retroviral elements that normally activate innate immune pathways (75, 76). Chromatin remodeling can promote epithelial–mesenchymal transition and shape an immune-excluded tumor microenvironment. The remodeling of the myeloid compartment, particularly through the expansion of CXCL8- and IL-8-driven neutrophils and myeloid-derived suppressor cells, produces a cytokine milieu rich in IL-10, TGF-β and VEGF, which impairs T-cell trafficking and effector function (77). Metabolic changes further contribute to resistance, since recurrent hypoxia enhances HIF-1α signaling, VEGF production and adenosine accumulation, whereas the selection of oxidative phosphorylation–high clones allows tumor cells to outcompete T cells for nutrients (78, 79).

In addition to tumor-intrinsic alterations and cellular components of the tumor microenvironment, soluble mediators, including chemokines and cytokines, play a critical role in shaping ICI response and resistance in dMMR–MSI-H CRC (80). Chemokine gradients such as CXCL9/CXCL10–CXCR3 signaling are associated with effective CD8^+^ T-cell recruitment and favorable ICI responses, whereas reduced expression of these chemotactic factors contributes to immune exclusion (81). Conversely, resistance is frequently accompanied by enrichment of immunosuppressive cytokines, including TGF-β, IL-10, and IL-6, which collectively impair dendritic cell activation, suppress effector T-cell function, and promote regulatory immune phenotypes (82). In parallel, myeloid-derived chemokines such as CXCL8 further reinforce neutrophil and MDSC recruitment, establishing a feed-forward immunosuppressive circuit that limits durable antitumor immunity (83).

## 4 Biomarker discovery

**Figure 4:**
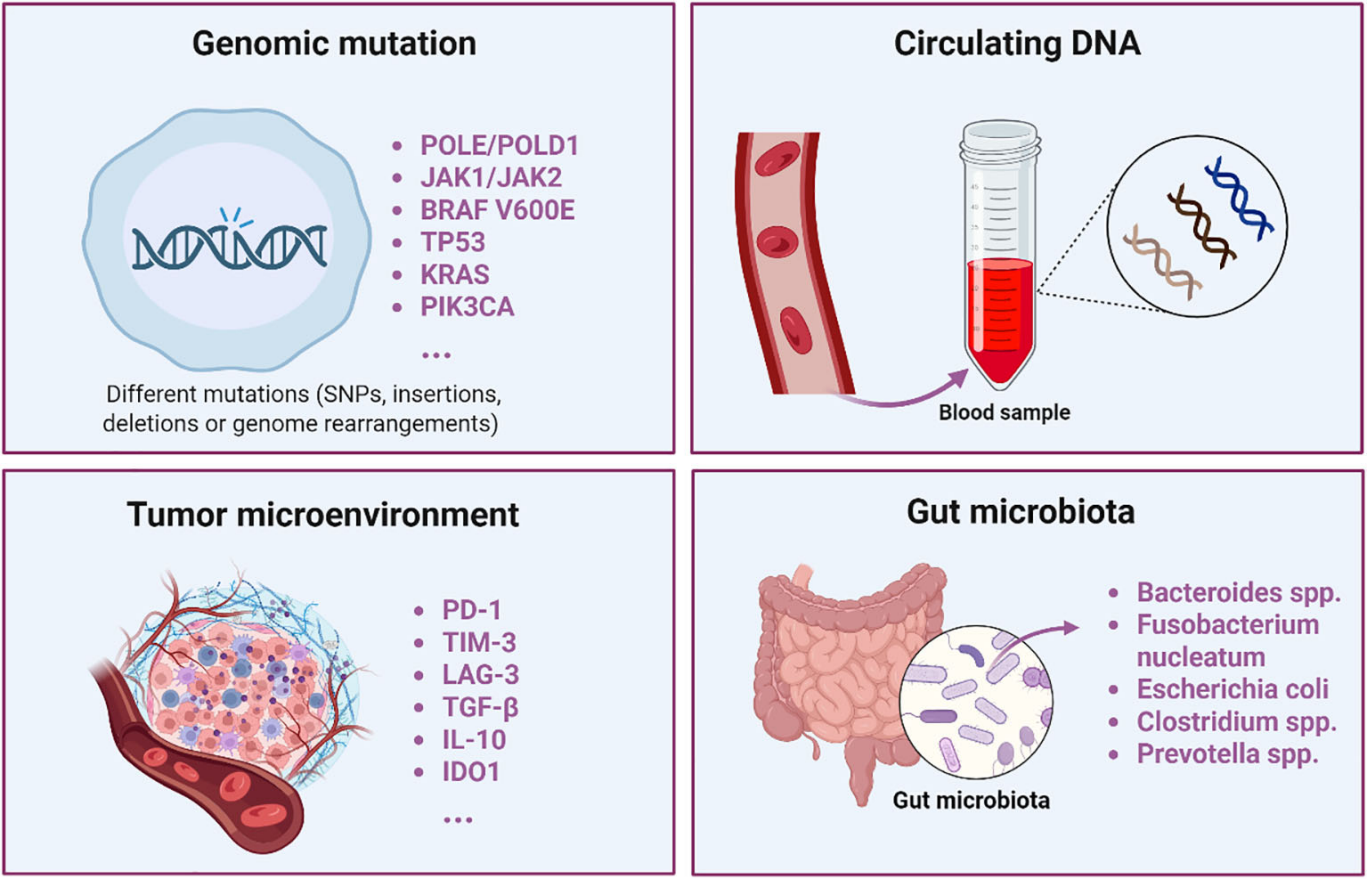
Factors associated with ICI resistance in dMMR–MSI-H CRC. This schematic summarizes the major categories of biomarkers implicated in primary and acquired resistance to ICI therapy in dMMR–MSI-H CRC. (1) Genomic alterations: mutations in POLE/POLD1, JAK1/JAK2, BRAF V600E, and TP53 can disrupt DNA repair fidelity, interferon signaling, or immune recognition, attenuating ICI efficacy. (2) Circulating DNA biomarkers: blood-derived circulating tumor DNA (ctDNA) reflects tumor mutational dynamics and emerging resistant clones. (3) Tumor microenvironmental factors, such as the upregulation of alternative immune checkpoints (PD-1, TIM-3, LAG-3), immunosuppressive cytokines (TGF-β, IL-10), and stromal remodeling, contribute to T-cell exhaustion and impaired cytotoxic function. (4) Gut microbial composition: Dysbiosis characterized by enrichment of *Fusobacterium nucleatum*, *Escherichia coli*, and Clostridium spp., along with a reduced abundance of beneficial taxa such as Bacteroides and Prevotella spp., modulates systemic immune responses and influences ICI efficacy. Collectively, these biomarkers highlight the complex interplay between tumor-intrinsic mutations, immune regulation, and microbial ecology in shaping ICI resistance in dMMR–MSI-H CRC.

Although dMMR–MSI-H CRC is characterized by a hypermutated phenotype and generally favorable responses to ICIs, approximately 20–40% of patients exhibit primary resistance or develop acquired resistance after initial benefit (84). This variability indicates that MSI status alone is insufficient for predicting durable response, underscoring the need for more refined genomic biomarkers (**Figure 4**).

### 4.1 Genomic mutations

Mutations in POLE and POLD1, which encode the catalytic subunits of DNA polymerases ϵ and δ, respectively, lead to defective proofreading and increased mutational burden (85, 86). However, excessive mutagenesis can also result in the selection of subclones with reduced immunogenicity or altered neoantigen quality, thereby diminishing immune recognition (87). Similarly, JAK1 and JAK2 loss-of-function mutations disrupt interferon-γ signaling, a critical axis for upregulating PD-L1 expression and sustaining cytotoxic T-cell activity (88). These alterations confer a state of “immunologic invisibility,” rendering tumor cells less responsive to ICI therapy. Moreover, activating BRAF V600E mutations and inactivating TP53 mutations remodel oncogenic signaling pathways, promoting epithelial–mesenchymal transition, stromal fibrosis, and immune exclusion (89). TMB is generally elevated in MSI-H cancers, but it varies substantially, and some studies suggest that patients with ultrahigh TMB or a greater proportion of clonal frameshift neoantigens experience deeper and more durable responses (90). The quality of neoantigens may be as important as their number, since strong predicted binding to patient-specific HLA molecules and consistent expression across metastatic sites appear to correlate more closely with clinical outcomes (91). Cooccurring genomic alterations also warrant attention. BRAF V600E mutations, which are enriched in sporadic MSI-H CRC due to MLH1 promoter methylation, confer a poor prognosis but have inconsistent predictive effects on ICI benefit (92). Other driver mutations, such as those in AT-rich interactive domain-containing protein 1A (ARID1A), may further refine sensitivity to ICIs in dMMR–MSI-H CRC. ARID1A is a key component of the SWI/SNF chromatin remodeling complex and plays an essential role in maintaining genomic stability and regulating transcriptional accessibility (93). Loss-of-function alterations in ARID1A have been associated with impaired DNA damage repair, increased TMB, and enhanced neoantigen generation, thereby reinforcing tumor immunogenicity. Mechanistically, ARID1A deficiency has been shown to promote interferon signaling and upregulate PD-L1 expression, while also facilitating increased infiltration and activation of CD8-positive T cells within the tumor microenvironment (94). In MSI-positive tumors, where baseline immunogenicity is already elevated, ARID1A mutations may further amplify these immune-responsive features and contribute to improved clinical benefit from PD-1 or PD-L1 blockade. Recent clinical and translational studies support this association, suggesting that ARID1A status may serve as a complementary biomarker for predicting immunotherapy responsiveness and may inform patient stratification in future studies (95). In addition, defects in antigen presentation also represent powerful predictors of resistance. Loss-of-function mutations or deletions in B2M, loss of heterozygosity at HLA class I loci, and alterations in peptide-loading components such as TAP1/2 and ERAP1/2 compromise CD8^+^ T-cell recognition (96). Similarly, inactivating mutations in interferon-γ signaling components impair the induction of antigen presentation and downstream immune effector programs, thereby reducing responsiveness to PD-1 blockade (97). The detection of such alterations in baseline tissue could help identify patients for whom monotherapy is unlikely to succeed.

### 4.2 Tumor microenvironment

The composition and spatial organization of the TME play critical roles in shaping the response to immune checkpoint inhibition in MSI-H CRC. Despite high neoantigen loads, many dMMR–MSI-H tumors exhibit a paradoxical lack of durable immune activation, driven by a progressive shift from an inflamed to an immunosuppressive TME (98, 99). The upregulation of alternative inhibitory receptors on exhausted CD8+ T cells limits effector cytokine production and cytotoxic function, even in the presence of active PD-1/PD-L1 blockade (100). Concurrently, tumor and stromal cells secrete immunosuppressive cytokines, which inhibit antigen presentation and Treg expansion and promote extracellular matrix deposition, which physically impedes lymphocyte infiltration (101, 102). Myeloid-derived suppressor cells and tumor-associated macrophages further reinforce this tolerogenic milieu by releasing arginase, IDO1, and adenosine, collectively dampening cytotoxic responses (103). Moreover, oncogenic mutations such as BRAF V600E or TP53 loss synergize with TGF-β signaling to sustain immune exclusion and epithelial–mesenchymal transition (104, 105). The result is a “cold” immune landscape refractory to ICI therapy, even in the molecular context of dMMR–MSI-H. High densities of intratumoral CD8^+^ cytotoxic T cells, particularly those with a tissue-resident memory phenotype marked by CD103, are strongly associated with favorable clinical outcomes (106). The presence of mature tertiary lymphoid structures (TLSs) within or adjacent to the tumor supports sustained local immune priming and has been linked to favorable responses (107, 108). Transcriptomic analyses further support these findings, showing that interferon-γ–driven immune signatures, T-cell-inflamed gene expression profiles, and elevated cytolytic activity scores correlate with better sensitivity to PD-1 blockade (109). In contrast, immunosuppressive TMEs characterized by the expansion of myeloid-derived suppressor cells, M2-polarized macrophages, and fibroblasts secreting TGF-β are associated with immune exclusion and a poor response (110). These features not only stratify patients by likely treatment outcome but also may guide rational combination strategies, including the addition of TGF-β inhibitors or antiangiogenic therapies. The spatial distribution of immune cells adds another layer of predictive information. Multiplex immunohistochemistry and spatial transcriptomics have shown that the close proximity of antigen-presenting dendritic cells and CD8^+^ T cells within the tumor core is associated with responsiveness, whereas peritumoral exclusion patterns often signal resistance (111). Single-cell RNA sequencing and T-cell receptor (TCR) profiling have also revealed functional heterogeneity within T-cell populations. Progenitor-exhausted T cells can be reinvigorated by PD-1 blockade, whereas terminally exhausted cells remain unresponsive, highlighting the importance of distinguishing between these subsets when assessing therapeutic potential (112, 113).

### 4.3 Circulating DNA

Blood-based biomarkers provide a minimally invasive way to monitor therapy and detect emerging resistance (114). Circulating tumor DNA (ctDNA) dynamics are especially promising. Unlike traditional tissue biopsies, ctDNA analysis enables real-time, noninvasive assessment of the evolving genomic landscape under therapeutic pressure (115, 116). Early clearance of ctDNA after ICI initiation correlates with objective response and durable benefit, whereas rising ctDNA may predict progression before radiographic evidence (117). Quantitative changes in ctDNA levels often mirror radiographic and clinical responses, allowing for early detection of disease progression or molecular relapse (118). These dynamic shifts provide insights into the temporal heterogeneity of ICI resistance, which may not be captured by static tumor sampling (119, 120). Furthermore, methylation and fragmentation patterns in circulating DNA may reflect epigenetic reprogramming within the tumor microenvironment, offering additional dimensions of immune escape biology (121). Integration of ctDNA sequencing with immunogenomic and transcriptomic data enhances the predictive precision of resistance monitoring (122, 123). In the context of ICI therapy, early clearance of ctDNA within weeks of treatment initiation has been strongly associated with objective radiographic responses, deep tumor regression, and prolonged progression-free survival (117). Conversely, rising ctDNA levels may serve as an early harbinger of disease progression. This lead time could be critical for prompt therapeutic adjustments, such as switching regimens or initiating combination strategies before clinical deterioration (118). Beyond dynamic quantification, ctDNA also facilitates molecular profiling in the blood, offering insights into TMB and the emergence of resistance-associated alterations (124, 125). Thus, ctDNA biomarkers represent a powerful tool for bridging molecular surveillance and clinical decision-making in the management of ICI resistance in dMMR–MSI-H CRC. Detecting these alterations noninvasively can guide the design of salvage strategies, for example, incorporating agents that bypass defective antigen presentation or restore interferon sensitivity.

### 4.4 Gut microbiome

The gut microbiome has emerged as a key systemic modulator of ICI efficacy in dMMR–MSI-H CRC, influencing both antitumor immune activation and the development of resistance (126). The microbial composition profoundly affects the systemic immune tone, shaping both antitumor immunity and resistance (127). Dysbiosis characterized by the enrichment of *Fusobacterium nucleatum* (*F. nucleatum*), *Escherichia coli* (*E. coli*), and Clostridium species is frequently associated with proinflammatory but immunosuppressive states that impair effective cytotoxic T-cell activation (128, 129). *F. nucleatum* can directly interact with immune cells via Fap2–TIGIT binding, inhibiting natural killer and T-cell activity, whereas certain *E. coli* strains produce genotoxins that promote DNA damage and tumorigenesis (130). In contrast, beneficial commensals such as Bacteroides and Prevotella support dendritic cell maturation, IL-12 production, and Th1-biased responses that increase ICI efficacy (131). Antibiotic exposure, low microbial diversity, and dietary factors can further disrupt this delicate balance, tipping the immune environment toward tolerance and resistance (132). Mechanistically, microbial metabolites modulate T-cell metabolism, cytokine production, and mucosal barrier integrity, thereby influencing tumor–immune crosstalk (133, 134). Restoring a favorable microbial composition through probiotics, prebiotics, or fecal microbiota transplantation (FMT) has shown promise in resensitizing tumors to ICIs in preclinical and early clinical studies (135). A growing body of evidence suggests that increased gut microbial diversity, often considered a hallmark of a healthy and resilient microbiota, is associated with improved clinical outcomes in patients receiving ICIs (136). Specific microbial taxa, most notably *Akkermansia muciniphila*, Bifidobacterium species, and certain Ruminococcaceae, have been linked to favorable therapeutic responses (137). These bacteria are thought to enhance host immune surveillance by promoting dendritic cell maturation, facilitating antigen presentation, and augmenting CD8^+^ T-cell infiltration into the tumor microenvironment. Short-chain fatty acids (SCFAs), such as butyrate, which are generated through microbial fermentation of dietary fiber, may also exert immunomodulatory effects by supporting regulatory T-cell homeostasis while enhancing effector T-cell function, thereby fine-tuning the balance between immune activation and tolerance (138, 139). In MSI-H CRC, where the tumor already harbors a high mutational burden and abundant neoantigens, a supportive microbiome may act as a catalyst that further amplifies immune recognition and cytotoxic activity (140). This synergistic effect could partly explain why some patients with otherwise similar genomic and immunologic profiles exhibit markedly different responses to ICIs. Importantly, the gut microbiome is not static; its composition can shift rapidly in response to diet, medications, illness, and other environmental factors (141). Its dynamic nature presents both a barrier and an opportunity. The issue stems from the possibility that a single baseline measurement may miss pertinent changes, while the promise is that intentional manipulation may be able to restore or improve ICI sensitivity (67). Ultimately, the gut microbiome represents a modifiable determinant of ICI efficacy that could complement existing genomic and immune-based biomarkers.

## 5 Promises and challenges of ICIs for dMMR–MSI-H CRC

Although dMMR–MSI-H status remains the strongest genomic predictor of durable benefit from PD-1/PD-L1 blockade in CRC, clinical experience has demonstrated that this biomarker does not guarantee response in all patients. Approximately 30–40% of patients show primary resistance, with no meaningful benefit from the outset, whereas others relapse after an initial phase of disease control (142). This heterogeneity underscores the complexity of immune–tumor interactions, even in hypermutated and immunogenic cancers. The incorporation of ICIs into first-line therapy, supported by multiple randomized trials, has significantly reshaped treatment paradigms. Notably, trials comparing nivolumab plus ipilimumab with nivolumab alone have shown improved progression-free survival for the combination in dMMR–MSI-H CRC (51, 143, 144). These findings, together with regulatory approvals and updates to clinical guidelines, confirm the feasibility and efficacy of combination strategies. However, the increased risk of immune-related adverse events introduces new challenges. Careful patient selection, improved tools to predict toxicity, and optimized management approaches are essential to balance efficacy with safety (145). As the field moves forward, research must focus on integrating rational combinations, overcoming drug resistance, personalizing treatment sequencing, and applying robust biomarker strategies to maximize benefit while minimizing harm. There are four prioritized strategies, accompanied by comprehensive molecular justifications, translational factors, and clinical trial ramifications, designed to enhance, prolong, and expand the advantages of ICI treatment in dMMR–MSI-H CRC.

### 5.1 Combination therapy

Dual or multicheckpoint blockade, such as combining PD-1 inhibition with CTLA-4, LAG-3, or TIM-3 inhibition, offers the potential to enhance antitumor immunity by acting at different stages of T-cell activation and effector function (146, 147). PD-1 blockade primarily restores the activity of exhausted effector T cells within the tumor microenvironment, whereas CTLA-4 blockade facilitates *de novo* priming of T cells in lymphoid tissues and broadens the T-cell repertoire (148, 149). In dMMR–MSI-H CRC, where abundant neoantigens are present, broadening the T-cell repertoire can be especially impactful, as it increases the probability of recognizing and targeting heterogeneous tumor clones. Clinical trials have already demonstrated that PD-1 plus CTLA-4 combinations improve PFS and response durability compared with PD-1 monotherapy in certain patient subsets (150). The benefit appears especially pronounced in patients with a high tumor burden, visceral metastases, or low baseline immune infiltration, clinical contexts in which PD-1 monotherapy may be insufficient to sustain antitumor activity. Nevertheless, combination therapy is accompanied by higher rates of grade 3–4 immune-related adverse events, making proactive toxicity management essential (151). Approaches to mitigate these risks include the use of steroid-sparing immunosuppressive agents, early identification of toxicities through biomarker monitoring and treatment de-escalation strategies to balance efficacy with safety. In the future, combination trials should incorporate translational endpoints such as pre- and on-treatment biopsies, immune infiltration profiling, TCR clonality analysis, and circulating tumor DNA kinetics to identify biological predictors of durable benefit and resistance (152). Such insights will help define which patients truly require upfront combination therapy and which can be effectively managed through sequential escalation. To ensure that enhanced efficacy does not come at the cost of long-term well-being, future studies should also embed quality-of-life assessments and patient-reported outcomes into trial designs.

### 5.2 Overcoming resistance

Both primary and acquired resistance to ICIs in dMMR–MSI-H CRC can be attributed to distinct tumor-intrinsic and microenvironmental mechanisms (153). Among tumor-intrinsic drivers, activation of the WNT/β-catenin signaling pathway promotes immune exclusion by blocking dendritic cell recruitment. Mutations in the JAK/STAT–interferon signaling axis diminish the ability of tumors to respond to interferon-γ, whereas defects in antigen presentation, including B2M loss or HLA class I alterations, render tumor cells invisible to cytotoxic T lymphocytes (154–156). Several therapeutic strategies are being developed to overcome these barriers. WNT pathway inhibitors may reverse immune exclusion, enabling dendritic cells and T cells to infiltrate the tumor (157). Engineered T-cell therapies and bispecific T-cell engagers can bypass defective antigen presentation by redirecting cytotoxic cells toward tumor-associated surface proteins (158). The TME also plays a central role in ICI resistance. Myeloid-driven immunosuppression, which is mediated by tumor-associated macrophages and myeloid-derived suppressor cells (MDSCs), is often reinforced by VEGF signaling, hypoxia, and metabolic suppressive pathways such as the adenosine axis and indoleamine 2,3-dioxygenase (IDO) activity (159). Together, these factors hinder T-cell trafficking, survival, and effector function. Combination strategies that pair ICIs with anti-VEGF agents, CSF-1R inhibitors, or metabolic modulators aim to remodel the microenvironment into a niche more permissive to T-cell activity (160).

To validate TME remodeling and guide rational combination designs, early-phase clinical trials should incorporate longitudinal immune profiling. Serial biopsies, multiplex immunohistochemistry, and single-cell RNA sequencing, in addition to systemic immune phenotyping, can reveal whether resistance mechanisms are present at baseline or emerge under selective pressure (161). Such approaches also help clarify whether resistance differs between metastatic sites within the same patient.

### 5.3 Increased immunogenicity

Although dMMR–MSI-H tumors are inherently rich in neoantigens, their immunogenicity can be further enhanced through additional therapeutic strategies. Cytotoxic chemotherapy and radiotherapy increase tumor antigen exposure by inducing immunogenic cell death, which releases damage-associated molecular patterns (DAMPs) that activate dendritic cells and promote cross-presentation (162, 163). Certain targeted therapies, including MEK inhibitors and PARP inhibitors, may also augment immune recognition by upregulating MHC class I expression or stimulating innate immune pathways such as cGAS–STING (164). The sequencing of these therapies is critical, as prolonged lymphodepletion could compromise the efficacy of checkpoint blockade. Short courses of lymphocyte-sparing chemotherapy or hypofractionated radiotherapy may provide the best balance. Localized interventions, such as stereotactic body radiation therapy or thermal ablation, can also trigger systemic immune responses when combined with ICIs, particularly in immune-cold metastatic lesions (165, 166). Personalized cancer vaccines and adoptive T-cell therapies represent another strategy for enhancing antitumor immunity. Neoantigen-based vaccines, which are designed using whole-exome sequencing and prediction algorithms to target clonal neoantigens, may help overcome tumor heterogeneity (167). Adoptive cell transfer approaches, including TCR-engineered T-cell and tumor-infiltrating lymphocyte therapies, provide a means to deliver large numbers of tumor-reactive lymphocytes directly to patients, even in the context of impaired antigen presentation (168).

Early-phase trials should incorporate comprehensive immune monitoring, including HLA typing, neoantigen clonality assessment, and functional persistence assays, to evaluate the quality and durability of immune responses. The establishment of centralized manufacturing platforms for personalized therapies may further streamline trial execution, reduce costs, and facilitate broader clinical application.

### 5.4 Biomarker-driven strategies

While the MSI-H status remains the most robust single biomarker for predicting ICI benefit, it alone cannot fully capture interpatient heterogeneity or reliably guide therapy escalation (46). More refined stratification can be achieved by integrating multiple biomarker layers, including genomic features, neoantigen burden and clonality, immune gene expression signatures, HLA genotype and integrity, and TME characteristics such as immune infiltration and myeloid composition (169, 170). Composite models that combine tumor genomics, immune gene expression, and circulating immune correlates hold the greatest promise for predictive precision. In addition to baseline predictors, dynamic early-response markers offer a critical opportunity to identify nonresponders within weeks of therapy initiation. These include ctDNA clearance, expansion of tumor-reactive T-cell clones in peripheral blood, and early radiographic changes (171). Adaptive trial designs can operationalize these insights by predefining escalation pathways. For example, patients showing suboptimal early responses on the basis of ctDNA kinetics could have additional checkpoint targets, anti-VEGF therapy, innate immune agonists, or cellular therapies added to their regimen (118). Conversely, patients with rapid and deep responses could be candidates for treatment de-escalation or fixed-duration therapy to reduce toxicity (172). To elucidate resistance mechanisms, longitudinal multiomics profiling—integrating paired tissue and ctDNA sequencing at baseline, during therapy, and at progression—is essential. Such efforts will map the evolutionary dynamics of antigen loss, immune exclusion, and pathway reactivation (173). Establishing shared biorepositories and harmonized analytic frameworks will enable meta-analyses and uncover rare but clinically actionable resistance patterns. Embedding translational endpoints such as ctDNA clearance and immune gene modulation as coprimary readouts can accelerate decision-making without waiting for long-term survival data. Finally, adopting standardized definitions of primary versus acquired resistance and aligning sampling schedules will enhance cross-trial comparability, facilitating both regulatory acceptance and the clinical implementation of adaptive precision strategies.

## 6 Conclusion

dMMR–MSI-H CRC exemplifies how tumor-intrinsic genomic features and the tumor–immune ecosystem jointly shape sensitivity to immune checkpoint blockade. The abundance of frameshift-derived neoantigens, high mutational burden, interferon-γ–enriched milieu, and pronounced lymphoid infiltration create highly favorable conditions for durable responses to PD-1/PD-L1- and CTLA-4-targeted agents. Compared with cytotoxic chemotherapy, both randomized and single-arm studies have established ICIs as transformative therapies in first-line and later-line settings, delivering durable remission and improving quality of life in a substantial subset of patients. Nonetheless, this paradigm also exposes a critical paradox, high immunogenicity does not uniformly translate into effective antitumor immunity. Approximately 20–40% of patients experience primary resistance, and others relapse after initial benefit. This disconnect reflects not a failure of neoantigen generation, but a breakdown in antigen processing, immune recognition, or effector function. Tumors may evade immune elimination through disruption of antigen presentation or interferon signaling, thereby rendering abundant neoantigens immunologically “invisible.” Concurrently, oncogenic signaling pathways can enforce immune exclusion, while myeloid-dominated suppressive niches, metabolic competition, and T cell dysfunction collectively blunt effective cytotoxic responses. In the acquired setting, immune editing and clonal selection further reshape the tumor landscape under therapeutic pressure, leading to the outgrowth of resistant subclones despite initial sensitivity. These observations underscore that MSI status, while necessary, is insufficient as a standalone predictor of ICI responsiveness. A more nuanced framework is required—one that integrates tumor-intrinsic features with dynamic immune contexture and systemic modulators such as the gut microbiome. Accordingly, emerging strategies are shifting toward biomarker-driven and adaptive therapeutic approaches, combining baseline genomic–immune profiling with real-time monitoring tools such as circulating tumor DNA and immune dynamics to identify nonresponders early and guide rational treatment escalation. Translationally, longitudinal tumor and blood sampling, coupled with single-cell, spatial, and multi-omics analyses, will be essential to map the evolution of resistance and to align mechanism-informed combination strategies. Ultimately, resolving the disconnect between immunogenicity and immune effectiveness will be key to extending durable benefit to a broader population of patients with dMMR–MSI-H CRC while minimizing unnecessary toxicity from empiric treatment intensification. Resistance arises through diverse mechanisms, including impaired antigen presentation or interferon signaling, oncogenic programs driving immune exclusion, myeloid-dominated immunosuppression, metabolic constraints within the TME, and systemic influences such as the gut microbiome. Multiple biomarkers and dynamic monitoring are needed to triage patients toward monotherapy, rational combinations, or alternative immune and cellular therapies. Clinically, the field is advancing toward biomarker-driven adaptive strategies that merge baseline genomic–immune profiling with real-time ctDNA and immune monitoring to identify nonresponders early and enable timely treatment escalation. This review integrates these dimensions into a unified mechanistic–translational framework, linking resistance biology with clinically actionable biomarker strategies to inform adaptive immunotherapy in dMMR–MSI-H CRC. Translationally, longitudinal tumor and blood sampling, single-cell and spatial profiling, and integrative multiomics analyses are essential to chart resistance evolution and align mechanism-specific interventions. Collectively, these approaches hold the promise of expanding the proportion of patients achieving long-term disease control while mitigating unnecessary toxicity from intensified regimens.
